# Treating Syrian refugees with diabetes and hypertension in Shatila refugee camp, Lebanon: Médecins Sans Frontières model of care and treatment outcomes

**DOI:** 10.1186/s13031-019-0191-3

**Published:** 2019-04-02

**Authors:** Maysoon Kayali, Krystel Moussally, Chantal Lakis, Mohamad Ali Abrash, Carla Sawan, Anthony Reid, Jeffrey Edwards

**Affiliations:** 1 Field mission, Médecins Sans Frontières, Operational Center Brussels, Shatila, Beirut, Lebanon; 2 Lebanon branch office, Médecins Sans Frontières, Beirut, Lebanon; 3 Lebanon mission, Médecins Sans Frontières, Operational Center Brussels, Beirut, Lebanon; 4 Division of Endocrinology, Diabetes, and Metabolism, Faculty of Medicine and Medical Sciences, University of Balamand, Beirut, Lebanon; 5 Operational Research Unit, Médecins Sans Frontières, Operational Center Brussels, Luxembourg City, Luxembourg; 6 Department of Global Health, University of Washington, Seattle, Washington USA

**Keywords:** Non-communicable disease, Refugee, Médecins Sans Frontières, Diabetes, Hypertension, Syrians

## Abstract

**Background:**

Médecins Sans Frontières (MSF) has been providing primary care for non-communicable diseases (NCDs), which have been increasing in low to middle-income countries, in the Shatila refugee camp, Beirut, Lebanon, using a comprehensive model of care to respond to the unmet needs of Syrian refugees. The objectives of this study were to: 1) describe the model of care used and the Syrian refugee population affected by diabetes mellitus (DM) and/or hypertension (HTN) who had ≥ one visit in the MSF NCD clinic in Shatila in 2017, and 2) assess 6 month treatment outcomes.

**Methods:**

A descriptive retrospective cohort study using routinely collected program data for a model of care for patients with DM and HTN consisting of four main components: case management, patient support and education counseling, integrated mental health, and health promotion.

**Results:**

Of 2644 Syrian patients with DM and/or HTN, 8% had Type-1 DM, 30% had Type-2 DM, 30% had HTN and 33% had DM + HTN. At intake, patients had a median age of 53, were predominantly females (63%), mostly from outside the catchment area (70%) and diagnosed (97%) prior to enrollment. After 6 months of care compared to intake: 61% of all patients had controlled DM (HbA1C < 8%) and 50% had controlled blood pressure (BP: < 140/90 mmHg) compared to 29 and 32%, respectively (*p* < 0.001). Compared to intake, patients with Type-1 DM reached an HbA1C mean of 8.4% versus 9.3% (*p* = 0.022); Type-2 DM patients had an HbA1C mean of 8.1% versus 9.4% (*p* = 0.001); and those with DM + HTN reached a mean HbA1C of 7.7% versus 9.0%, (*p* = 0.003). Reflecting improved control, HTN patients requiring ≥3 medications increased from 23 to 38% (p < 0.001), while DM patients requiring insulin increased from 21 to 29% (p < 0.001). Loss-to-follow-up was 16%.

**Conclusions:**

The MSF model of care for DM and HTN operating in the Shatila refugee camp is feasible, and showed promising outcomes among enrolled individuals. It may be replicated in similar contexts to respond to the increasing burden of NCDs among refugees in the Middle-East and elsewhere.

## Background

Non-communicable diseases (NCDs) are leading causes of morbidity and mortality globally [1]. Their prevalence and burden are projected to continuously increase, with a more pronounced increase in low to middle-income countries such as Lebanon [2, 3], where an estimated 1.5 million Syrians have been displaced following the onset of the Syrian war in 2011 [4], creating a crisis situation in the country and a huge burden on the Lebanese health care system including that for NCDs [5]. In the refugee population, NCDs can be aggravated by disruption and shortage of medication supplies, lack of access to care and acute exacerbations related to these challenges [6, 7].

In Lebanon, a country characterized by a highly privatized and expensive health care system, there are significant financial barriers to healthcare for the vulnerable refugee population [8], many of whom have pre-existing NCDs [9–11]. Although health care actors are present in Lebanon to respond to the needs of the refugee population, very little is done for NCDs which is still a high burden within this population. Poverty, often associated with refugee status, can contribute to the worsening of NCDs [12, 13]. Additionally, NCDs often lead to lower earning potential, decreased quality of life, increased morbidity and mortality, and a perpetuation of the poverty cycle [14, 15]. The prevalence of diabetes mellitus (DM) and hypertension (HTN) in the Syrian refugee population living in Lebanon has been recently estimated to be of 9.9 and 20.5%, respectively [9].

In order to meet the needs of the refugee population in the country, Médecins Sans Frontières (MSF) has been providing NCD care since 2013 at the primary healthcare level in the Shatila refugee camp, south of the capital Beirut. The model of care that MSF offers is a comprehensive package adapted to the context of the humanitarian crisis setting. Even though an innovative approach to the model of care was used, it was a significant challenge to successfully reach the target outcome goals for these diseases.

While a few studies and assessments have been published on the prevalence of NCDs among the Syrian refugees in Lebanon [9–11], to our knowledge, none has described what model of care is used to serve this population, nor the population’s characteristics nor the outcomes of care. Moreover, a recent systematic review looking at the evidence available on the effectiveness of interventions for NCDs in humanitarian contexts highlighted the enormous gap and limited quality of evidence on this topic [16]. These gaps included: the feasibility of NCD interventions in crisis settings, transparency in reporting of follow-up periods, full descriptions of the limitations of the studies as well as inadequacy of outcomes, most of which were self-reported [16]. Additionally, none of the studies included in this analysis were conducted in a refugee setting in Lebanon.

Therefore, the aim of this study was to address these gaps and add to the scarce evidence available by describing the model of care utilized to manage the Syrian refugee population affected by DM and/or HTN followed at the MSF NCD clinic in Shatila, Beirut, Lebanon, as well as to describe patient characteristics and assess treatment outcomes.

## Methods

### Study design

This was a descriptive retrospective cohort study carried out using routinely collected program data.

### MSF NCD program

The NCD program is part of an integrated primary care program that MSF started in 2013 in Shatila, a refugee camp located in southern Beirut in Lebanon and home to an estimated 40,000 population, half of whom are refugees who have fled the war in Syria since 2011. Living conditions in the camp are challenged by poor infrastructure, insecurity, lack of health care coverage, and a significant level of poverty.

The MSF NCD program in Shatila serves the refugee and vulnerable host communities affected by the following NCDs: diabetes, hypertension, other cardiovascular diseases (CVD) -defined as one of the following: ischemic heart disease, heart failure, transient ischemic attack, cerebrovascular accident, or peripheral arterial disease-, chronic obstructive pulmonary disease, asthma, epilepsy, and hypothyroidism, with no age restriction. In 2017, the model of care for patients with DM and HTN was based on four main components: case management, patient support and education counseling (PSEC), integrated mental health, and health promotion (Table 1).

**Table 1:** MSF diabetes and hypertension model of care, Shatila primary care clinic, Beirut, Lebanon, 2013–2017

Model of care component	Details		
Case-Management	Nurse’s consultation consists of checking vital signs, fasting blood glucose and blood pressure measurements done at every consultation for DM and HTN patients. In addition, the NCD nurse checks vital signs for all scheduled NCD patients including the ones who present to see the doctor		
	Doctor’s consultation is provided by trained general practitioners. Patients are not seen by specialist doctors at the MSF clinic at any time. If and when advised by the treating doctor, a patient with DM and/or HTN might be referred to a specialist as clinically indicated. All new patients are diagnosed by the doctors following MSF guidelines. DM is diagnosed with: a fasting plasma glucose level of ≥ 126 mg/dl (≥ 7 mmol/L) and clinical symptoms at first visit, or at ≥2 consecutive visits without clinical symptoms; or a random glucose level of ≥200 mg/dl (≥ 11.1 mmol/L) at ≥ 2 consecutive visits; or an HbA1C of ≥ 6.5%. HTN is diagnosed with: a SBP > 140 mmHg and/or a DBP > 90 mmHg at three clinical visits over 3 weeks; or a SBP > 180 mmHg and/or a DBP > 110 mmHg at first visit; or a SBP from 140 to 159 mmHg and/or DBP from 90 to 99 mmHg with a cardiovascular risk > 20% [WHO/ISH risk prediction chart] or a co-morbidity (cardiovascular disease, chronic kidney disease, DM).		
	NCD nurse and doctor consultations are provided interchangeably based on the below schedule:		
		NCD nurse	Doctor
	New patients with DM and/or HTN	None	Every 1 to 2 months until they are controlled
	Uncontrolled HTN	None	Every 1 to 2 months
	Improving uncontrolled DM	Every 3 to 6 months	Every 2 to 4 months
	Controlled DM or HTN	Every 6 months	Every 6 months
	Patients with exacerbations	None	As needed
	Drugs and glucometers are provided and renewed by the MSF pharmacist. Glucometers are provided for patients on insulin and pregnant women.		
	Primary laboratory investigations carried out are:		
	* HbA1C every 3 months for uncontrolled DM patients and every 6 months for controlled DM patients.		
	* Total cholesterol, creatinine, and urine dipstick at enrollment (new patients) and annually or as needed.		
	All laboratory tests, including HbA1C were done in the same external quality assured reference laboratory		
Patient support and education counseling (PSEC)	PSEC is provided only for DM patients. HTN patients are not included in the PSEC due to a limited program capacity forcing prioritization of resources.		
	Patients are referred to the PSEC by doctors. Referral is based upon the doctor’s clinical judgment for patients with uncontrolled DM who are willing to be supported in self-managing their disease, while all the newly diagnosed DM patients and the pregnant women are referred.		
	PSEC services are provided one-on-one by trained health promotion personnel in the same primary healthcare center.		
	The PSEC package includes education support and counseling on the disease and its complications, adherence to medications, self-monitoring of blood glucose and lifestyle habits with diet instructions, the latter being the first-step considered in the case management of DM patients besides introducing medications. It is a package adapted to the resources available for refugees.		
Mental health	Mental health services are integrated in the NCD model of care.		
	Patients are referred by the doctors or by the PSEC personnel based on clinical judgment.		
	Mental health sessions are provided by psychologists in the same primary healthcare center.		
Health promotion	Sessions are provided systematically and on a regular basis in waiting areas in groups by health promoters. They tackle general topics related to DM and HTN awareness.		

*DBP* diastolic blood pressure, *DM* diabetes mellitus, *HTN* hypertension, *ISH* international society of hypertension, *MSF* Medecins Sans Frontieres, *NCD* non-communicable diseases, *PSEC* Patient Education Support and Counseling, *SBP* systolic blood pressure, *WHO* world health organization

The model developed by MSF is an evolving, dynamic model, continuously adapting with the increasing knowledge of the context and the community cared for. For instance, provision of glucometers and PSEC were introduced mid-2016. Also in December 2016, task-shifting was introduced whereby patients with DM and/or HTN are seen interchangeably by the NCD nurse and doctor depending on criteria related to patients’ disease status (Table 1). Two doctors and one nurse average 35 and 32 NCD consultation per day each respectively. This task-shifting and regular adaptation of the model resulted in a comprehensive, multidisciplinary approach to the management of DM and HTN, giving more time to explore patients’ needs, concerns and understanding of their condition. All services, including medications provided under this model of care, are free of charge.

Referrals to secondary and specialized care for DM and HTN complications are not an integral part of the model and only emergency cases are referred for hospitalisation due to limited capacity and resources. Therefore, disease complications such as macro- or micro-vascular complications are not addressed as part of our model. However, social workers are available to provide guidance to the patients in need of secondary referrals and to communicate with different heath care stakeholders providing these services.

The protocols used in the management of DM and HTN patients were drafted by MSF based on guidelines of the WHO, the National Institute for Health and Care Excellence (NICE), and the European Society of Hypertension and Cardiology [17–19]. These MSF protocols and the monitoring of DM and HTN patients are continuously updated and adapted to respond to the programmatic and situational challenges, financial barriers faced by the community served and the local context of the ongoing humanitarian crisis. In fact, these patients struggle to meet their basic daily needs, have limited resources preventing them from following healthy life style habits, have a low level of healthcare literacy, and frequently have to relocate their place of residence. In addition, some face security challenges related to their legal status preventing them from moving freely and attending the clinic.

In order to account for these challenges, visits were made comprehensive to include consultation, laboratory tests, drug delivery and PSEC all during the same day; the number of follow-up visits was reduced; longer supplies of medications were given (3 months for controlled patients); and task-shifting reduced the waiting time for patients. Clinical adaptations included setting the HbA1C target to be < 8% rather than < 7% (international guidelines), widening of the interval between HbA1C measurements (every 6 months rather than 3 months for controlled DM patients), and simplifying the list of medications by adopting the essential list of medications of the Lebanese Ministry of Health [20]. For instance, only two oral anti-diabetic agents were provided for Type-2 DM: metformin and glibenclamide.

Three types of insulin were available: pre-mixed (intermediate and short-acting insulin) 70/30 type, short-acting (regular) insulin and intermediate acting insulin. Newer oral anti-diabetic medications (DPP-4 inhibitors, SGLT-2 inhibitors, GLP-1 agonists) as well as long-acting (basal) and rapid-acting insulins were not available.

Antihypertensive medications included a thiazide diuretic (hydrochlorothiazide), angiotensin converting enzyme (ACE) inhibitors (enalapril, ramipril, and captopril), an angiotensin-2 receptor blocker (losartan), a beta-blocker (bisoprolol), a calcium channel blocker (amlodipine), as well as other diuretics (furosemide and spironolactone). Methyldopa was prescribed for pregnant hypertensive women when indicated.

As of the end of 2017, the NCD program was providing services for almost 3500 patients with an average of 166 new cases and 1800 consultations/month during 2017. Of all NCD patients on follow-up in 2017, 76% (*n* = 2644) were Syrian patients with DM and/or HTN. Although the NCD program is meant to serve the catchment area of Shatila and its surroundings, including host and refugee communities, the majority of the patients presenting to the clinic were Syrian refugees coming from outside of the catchment area.

### Study population

This descriptive cohort included all Syrian patients: 1) with DM (types 1 and 2) or HTN, 2) who were enrolled in the MSF NCD program at any time up to 31 December 2017, and 3) who had at least one visit to the MSF Shatila clinic in 2017. For the treatment outcome analysis, patients had to: 1) be enrolled as of 30 June 2016 and be in the program for at least 6 months, 2) have ≥ two HbA1C tests for patients with DM and ≥ two visits with blood pressure (BP) measurements for patients with HTN recorded during their follow-up, with 3) the second HbA1C and/or BP recorded within 6 months of the first 1 ± 2 months. This specific sample of patients enrolled in the program and with a follow-up period restricted to ≥ 6 months and ≤ 18 months, was selected for the assessment of the treatment outcome to permit the analysis in a period where the program and the model of care were applied consistently with no major changes, which allows for an optimal reflection on the care delivered under these conditions.

### Data sources

Data was retrieved from the electronic District Health Information System, version 2 (DHIS2) that captured patient-specific data. Trained personnel retrospectively recorded NCD program data extracted from the paper-based medical patients’ files in a standardized fashion into the DHIS2 system on a daily basis. This electronic system is currently used at country level in Lebanon for monitoring of other health services [21]. Variables included socio-demographic characteristics (age, gender, nationality, place of residence), program and clinical variables at first visit [follow-up time on the program, previously diagnosed disease, CVD as a co-morbidity, HBA1C, systolic blood pressure (SBP), diastolic blood pressure (DBP), number of medications and insulin use (at first and last visit), and clinical diagnosis]. Missing and outlier data were as much as possible verified from the source paper-based files when accessible.

### Treatment outcomes

Outcomes of DM and HTN treatment were assessed after 6 months of care from enrollment. International consensus guidelines recommend a target HbA1C of < 7% for the majority of patients’ groups, and we aimed at providing our vulnerable population with the same standard of care. However, we were obliged to set an HbA1C target that is as close as possible to the international standards, taking into account the contextual challenges faced by this population in the access to care, medications and follow-up. Therefore, controlled diabetes was defined as an HbA1C value of < 8%. Controlled hypertension was defined as a BP < 140/90 mmHg. Six months HbA1C and BP values were compared to the first recorded (baseline) HbA1C and BP values for the same patients. The first HbA1C values recorded might not have been the ones collected at the enrollment visit. Therefore, patients were excluded from the outcome analysis if a first recorded HbA1C was dated > 3 months after the enrollment date. Patients who did not present at their scheduled appointment within a 6 months’ time period were defined as lost to follow-up (LTFU).

### Statistical analysis

Four main populations were defined based on their disease: patients with Type-1 DM only, patients with Type-2 DM only, patients with HTN only and patients with both DM (Types-1 or 2) and HTN. Descriptive statistics were used to describe the characteristics of the study population upon enrollment. Variables with > 5% of their data missing were excluded.

For the treatment outcomes of DM patients (with or without HTN), the mean HbA1C at 6 months of care after enrollment was calculated and compared to the mean HbA1C at the first recorded visit. In addition, the proportion of patients with HbA1C < 8% at 6 months was compared to that at the first recorded visit. HbA1C values < 4% were excluded. For patients with HTN (with or without DM), the proportion of those with a BP < 140/90 mmHg at 6 months was compared to the same proportion at first recorded visit. A range of ± 2 months was applied to outcome periods since many patients at the NCD clinic in Shatila did not present at the exact date of their scheduled appointment; and also to account for possible delays in receiving the laboratory test results.

Wilcoxon signed rank tests or paired t-tests, and McNemar tests were used to compare the changes in the means and proportions. A *p*-value < 0.05 was considered statistically significant and 95% confidence intervals were used. Data was exported from DHIS2 into Excel (Microsoft Corp, Redmond, Washington, USA) for data cleaning and analyzed using SPSS (USA, IBM corporation software, version 20).

## Results

### Patient characteristics

**Fig. 1:**
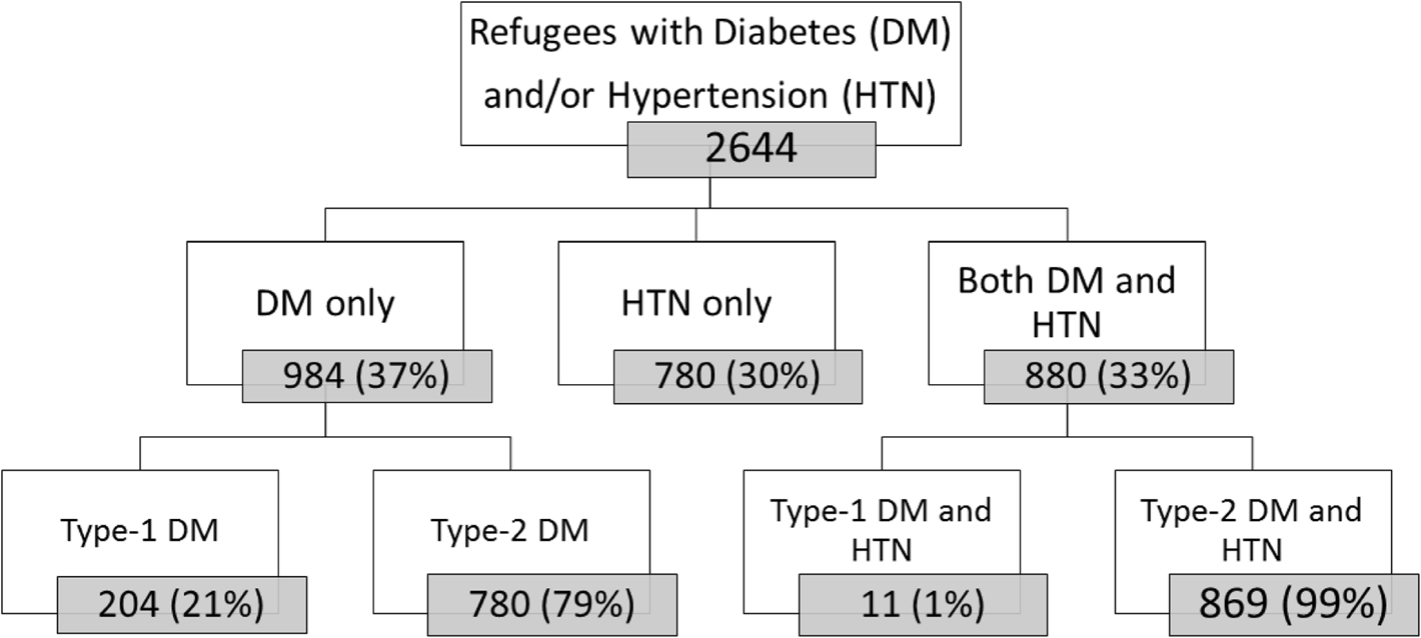
Flow chart, Syrian patients with diabetes and hypertension, Shatila primary care clinic, Beirut, Lebanon 2013–2017

Of the total cohort, 2644 (76%) patients with DM or HTN had at least one visit to the MSF Shatila clinic in 2017 and were Syrian refugees. The majority (37%, *n* = 984) only had DM at their first visit, with Type-2 being the most prevalent (79%, *n* = 780) among them; 30% (n = 780) had HTN only and 33% (*n* = 880) had both DM and HTN as co-morbidities at their first visit (Fig. 1).

All patients, independent of their diagnosis, had a median age of 53 [Interquartile Range (IQR):45–61] and were predominantly females (63%, *n* = 1666). However, patients with Type-1 DM had a lower median age of 22 (IQR: 12–32), and were mostly males (57%, *n* = 116) (Table 2). The majority of patients (70%, *n* = 1789) came from outside of the catchment area. Almost all patients (97%, *n* = 2566) had been previously diagnosed. Among all patients, 14% (*n* = 375) had a cardiovascular comorbidity at their first visit. This figure was highest (20%, *n* = 158) amongst patients with HTN only compared to the other groups.

**Table 2:** Characteristics of Syrian patients with diabetes and hypertension, Shatila primary care clinic, Beirut, Lebanon, 2013–2017

Characteristics at first visit	DM-1 Only (*n* = 204)	DM-2 Only (*n* = 780)	HTN Only (*n* = 780)	DM + HTN (*n* = 880)	All patients (*N* = 2644)
Age - year *(median, IQR)*	22 (12–32)	51 (44–58)	54 (47–62)	57 (51–63)	53 (45–61)
Age categories - year - *n (%)*					
<18 y	75 (36)	1 (< 1)	2 (< 1)	0 (0)	78 (3)
> = 18- < 40 y	112 (55)	113 (14)	79 (10)	36 (4)	340 (13)
> = 40- < 60y	16 (8)	506 (65)	454 (58)	513 (58)	1489 (56)
> = 60 y	1 (< 1)	160 (20)	245 (31)	331 (38)	737 (28)
Gender - *n (%)*					
Female	88 (43)	459 (59)	534 (68)	585 (66)	1666 (63)
Male	116 (57)	321 (41)	246 (32)	295 (34)	978 (37)
Place of residency - *n (%)*					
In catchment area	55 (28)	218 (29)	271 (36)	221 (26)	765 (30)
Outside catchment area	143 (72)	539 (71)	484 (64)	623 (74)	1789 (70)
Previously diagnosed - *n (%)*					
Yes	199 (98)	733 (94)	760 (97)	874 (99)	2566 (97)
No - newly diagnosed	5 (2)	47 (6)	20 (3)	6 (<1)	78 (3)
Cardiovascular co-morbidity^a^					
Yes	2 (< 1)	75 (10)	158 (20)	140 (16)	375 (14)
No	202 (99)	705 (90)	622 (80)	740 (84)	2269 (86)
HbA1C - % *[mean (SD)]*	9.9 (2.1)	8.9 (2.1)	NA	8.7 (2.0)	9.0 (2.1)
Blood pressure^b^ - mmHg [(mean (SD)]					
Systolic blood pressure	NA	NA	142 (25)	139 (22.4)	141 (23.7)
Diastolic blood pressure	NA	NA	87 (14.4)	84 (12.8)	86 (13.7)
Number of prescribed medications^b^					
1	NA	NA	249 (33)	335 (40)	584 (36)
2	NA	NA	321 (42)	332 (40)	653 (41)
> = 3	NA	NA	193 (25)	168 (20)	361 (23)
Insulin use^c^					
Yes	204 (100)	73 (9)	NA	117 (13)	394 (21)
No	0 (0)	705 (91)	NA	751 (87)	1456 (79)
Characteristics at last visit					
Number of prescribed medications^b^					
1	NA	NA	167 (22)	219 (26)	386 (24)
2	NA	NA	296 (39)	325 (38)	621 (38)
> = 3	NA	NA	300 (39)	310 (36)	610 (38)
Insulin use^c^					
Yes	204 (100)	141 (18)	NA	190 (22)	535 (29)
No	0 (0)	639 (82)	NA	690 (78)	1329 (71)
Follow-up period while in the program - months *(median, IQR)*	14 (6–23)	11 (4–19)	13 (5–25)	16 (6–26)	13 (5–24)
Lost to follow-up - *n (%)*	18 (9)	133 (17)	136 (17)	139 (16)	426 (16)

*DM-1* type-1 diabetes, *DM-2* type-2 diabetes, *HTN* hypertension, *IQR* interquartile range, *NA* not applicable, *SD* standard deviation

^a^Cardiovascular co-morbidity is defined as one of the following: ischemic heart disease, heart failure, transient ischemic attack, cerebrovascular accident, or peripheral arterial disease

^b^Calculated for patients with hypertension; 43 patients (2.6%) had this data missing in their files and were excluded from the calculation

^c^Calculated for patients with diabetes

Among Type-2 DM patients, there were 73 patients (9%) on insulin at their first visit compared to 18% (*n* = 141) at their last visit in addition to one oral anti-diabetic medication (Table 2).

Patients with HTN presented with a mean SBP of 141 mmHg [Standard deviation (SD), 23.7] and a mean DBP of 86 mmHg (SD, 13.7) at their first recorded visit. Twenty-three percent (*n* = 361) were prescribed three or more anti-hypertensive medications at their first visit compared to a statistically significant increase to 38% (*n* = 610, *p* < 0.001) at their last visit.

The median follow-up period on the program was 13 months with 25% of the patients being on follow-up for more than 24 months. Patients with DM and HTN had a higher median period of follow-up (16 months) compared to the other categories, Type-2 DM being the lowest (11 months). Overall, LTFU was 16% (*n* = 426) for all patients over 6 months follow-up. Type-1 DM patients were the most retained on care (LTFU 9%, *n* = 18) compared to the other categories (Table 2).

### Patient outcomes

**Fig. 2:**
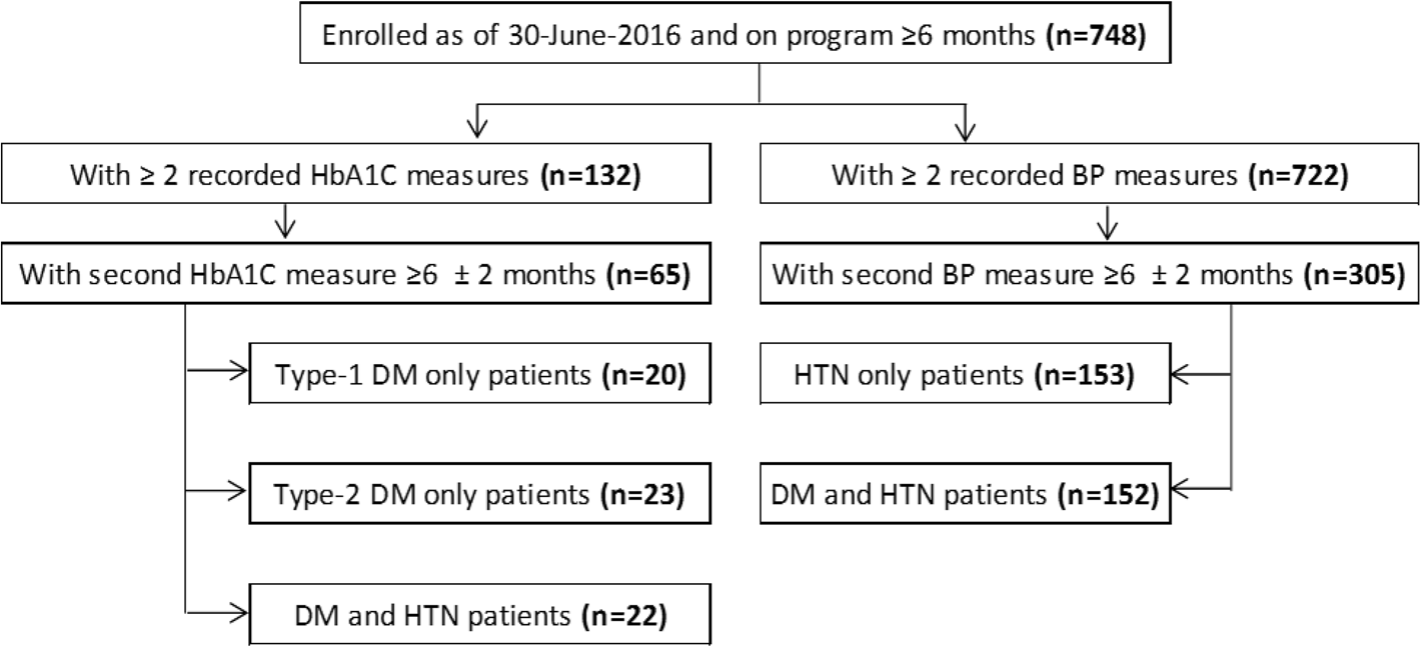
Flow chart, Syrian patients with diabetes and hypertension, outcome analysis, Shatila primary care clinic, 2016–2017. DM diabetes mellitus, HTN hypertension

Out of a total of 748 patients who were followed for at least 6 months till 31 December 2017, 65 (9%) and 305 (41%) patients met the inclusion criteria for DM and HTN respectively, and were included in the outcome analysis. Patients who did not meet the inclusion criteria (*n* = 683 and *n* = 443 for DM and HTN respectively) were mainly patients who did not have ≥ 2 HbA1C or BP recorded measures during the study period. Reasons could be possibly due to values not recorded in the electronic files or to missed appointments. The analysis was performed on 20 patients with Type-1 DM only, 23 patients with Type-2 DM only and 22 patients who had DM and HTN together; and on 153 patients with HTN only and 152 patients with DM and HTN for the HTN outcomes (Fig. 2).

Overall, 61% of patients with DM only and patients with DM and HTN (*n* = 40) had an HbA1C < 8% at 6 months of care compared to 29% (*n* = 19) at baseline (*p* < 0.001). A statistically significant increase in the proportion of patients with HbA1C < 8% at 6 months compared to baseline was also observed in all subgroups of patients (Table 3). The majority of patients with Type-1 DM (55%, *n* = 11) had an HbA1C < 8% at 6 months of care and the mean HbA1C at 6 months was 8.4% compared to a baseline mean of 9.3% (*p* = 0.022). In Type-2 DM patients, HbA1C mean at baseline was 9.4% compared to 6 months follow-up at 8.1% (*p* = 0.001), see Table 3. Patients with DM and HTN presented with the lowest baseline HbA1C mean (9.0%) and reached the lowest HbA1C target at 6 months (7.7%) compared to the others (*p* = 0.003), see Table 3.

**Table 3:** Six-month diabetes treatment outcome in Syrian patients, Shatila primary care clinic, Beirut, Lebanon, 2016–2017

	Baseline HbA1C	Six month HbA1C	*p*-value^a^
HbA1C % - [mean (SD); min-max]			
Type-1 DM (n = 20)	9.3 (1.8); 6.0–12.6	8.4 (1.4); 6.7–12.3	0.022
Type-2 DM (n = 23)	9.4 (2.5); 5.8–14.4	8.1 (1.8); 5.7–12.7	0.001
DM and HTN (n = 22)	9.0 (2.0); 5.7–12.9	7.7 (1.6); 5.5–11.8	0.003
HbA1C < 8% - [n (%)]			
Type-1 DM (n = 20)	4 (20)	11 (55)	0.016
Type-2 DM (n = 23)	8 (35)	15 (65)	0.016
DM and HTN (n = 22)	7 (32)	14 (64)	0.039

*DM* diabetes mellitus, *HTN* hypertension, *SD* standard deviation

^a^*p*-value <0.05 is statistically significant; Wilcoxon signed rank tests for mean and McNemar tests for proportions

Forty-nine percent (*n* = 75) of patients with HTN only reached the target BP at 6 months of care, compared to 27% (*n* = 42) at baseline (p < 0.001); while 52% (*n* = 79) of those with HTN and DM reached the target BP at 6 months versus 36% (*n* = 55) at baseline (*p* = 0.006) (Table 4). Patients with HTN only presented with a higher uncontrolled baseline SBP and DBP mean (145 and 89 mmHg) compared to the patients with DM and HTN (140 and 86 mmHg).

**Table 4:** Six-month hypertension treatment outcome in Syrian patients, Shatila primary care clinic, Beirut, Lebanon, 2016–2017

	Baseline BP	Six month BP	*p*-value^a^
SBP mmHg - [mean (SD); min-max]			
HTN (n = 153)	145 (23.9); 100–220	129 (18.7); 100–190	<0.001
HTN and DM ( n= 152)	140 (22.3); 80–210	132 (23.2); 80–260	<0.001
DBP mmHg - [mean (SD); min-max]			
HTN (n = 153)	89 (15.0); 60–130	83 (10.8); 50–110	<0.001
HTN and DM (n = 152)	86 (12.4); 50–110	82 (15.0); 50–180	0.011
Controlled BP - [n (%)]			
HTN (n = 153)	42 (27)	75 (49)	<0.001
HTN and DM (n = 152)	55 (36)	79 (52)	0.006

*DBP* diastolic blood pressure, *DM* diabetes mellitus, *HTN* hypertension, *SBP* systolic blood pressure, *SD* standard deviation

^a^*p*-value <0.05 is statistically significant; paired t-tests for mean and McNemar tests for proportions

## Discussion

To our knowledge, this is the first study describing a program of treatment for DM and HTN among Syrian refugees in Lebanon. It brings new evidence on the effectiveness of the program and treatment of DM and HTN patients in the humanitarian context of the Syrian crisis. Others have looked at the prevalence and access to NCD care in the region and Lebanon [9–11, 22], but there have been no description of the characteristics of this population, or of treatment outcomes.

This study also described the feasibility of a comprehensive, dynamic and multidisciplinary DM and HTN model operating at a primary care level that was continuously adapted to account for the programmatic and contextual challenges faced by the patients. It attracted many refugees to care, even from outside the catchment area, and demonstrated achievable outcomes deemed successful in a challenging crisis context and over a relatively short period of time (6 months). Additionally, it showed that the use of HbA1C, recommended as the method of choice for monitoring DM as compared to blood glucose measurements [23], is feasible in this context.

The characteristics of the Syrian refugees’ population seen in our clinic are comparable to the profile of patients with DM and/or HTN seen elsewhere in the Middle-East in terms of age and gender [22–25]. The fact that most of the patients came from outside of the catchment area may be explained by MSF’s successful and attractive comprehensive model of care that allowed patients to see the doctor, get their medications and glucometer if needed, carry out laboratory tests and receive education - all during the same visit and free of charge. This was especially important for patients with significant financial constraints. In that sense and unlike other NCD programs targeting different refugee contexts [24–26], the MSF model of care was adapted in a way to reduce the burden of visits on the patients. For instance, the model reduced the frequency of the visits to the minimum possible, decreased their length and avoided patients having to move outside of the clinic for tests or drugs, while still achieving high quality care.

While most patients at the MSF clinic had a previously diagnosed disease, which is an indicator of a relatively functional healthcare system in Syria [27], many were found to have uncontrolled DM and/or HTN at baseline. This implies that although aware of their disease, they had challenges accessing health care in Lebanon [5, 9]. For instance, even though all of the patients with Type-1 DM were on insulin when they were seen for their first visit to our program, the mean of their baseline HbA1C was high (9.3%). This is likely because they could not afford to pay for insulin, glucometers or test strips that are needed to monitor blood glucose levels.

The high baseline HbA1C mean of Type-2 DM patients could be an indicator of lack of access to adequate medications, regular medical follow-up and/or poor dietary options. Reasons could be financial barriers, lack of knowledge of the availability of services, or lack of medical awareness of the severity of their disease and the impact of a non-regular follow-up on long term complications. Despite a statistically significant drop in HbA1C in Type-1 DM patients after 6 months of care, they still had, on average, an uncontrolled HbA1C and the magnitude of the drop was still less than what was observed in Type-2 DM patients. This is to be expected given the particular physiological, psychosocial and behavioral challenges [28, 29] related to the Type-1 DM and patients’ specific circumstances, making them more difficult to control. In fact, they require a more frequent insulin regimen, with careful glucose monitoring and represent a younger population with a higher likelihood of non-compliance [30].

Studies looking at DM outcomes in Palestinian refugees have also shown that the proportion of patients with a controlled DM is higher in patients with Type-2 DM or DM and HTN compared to Type-1 DM [26], consistent with our findings. In addition, although there is no convincing evidence supporting improved efficacy with modern insulin [31, 32], if longer acting insulin were available in our clinics, they may have helped in better controlling challenging cases of Type-1 DM by improving compliance.

While it is expected that patients with DM and HTN would have more difficulties in achieving good outcomes, they actually reached a lower target HbA1C compared to the others. In this population, 20% were prescribed three or more drugs for their HTN at their first visit and 13% were already on insulin. These results would suggest that this patient group was more aware of their co-morbidities and had received better management at home and/or more health education over the years. This is the opposite of what one might think of in a crisis context, where polymedicated patients would more likely undergo treatment interruption, and suboptimal compliance. Fortunately, our results showed that these patients arrived better controlled for their DM and HTN and achieved a lower HbA1C target compared to other groups. This finding suggests that aiming for a lower HbA1C target in this population may be possible if clinically indicated. As in our study, another in a Palestinian refugee community showed that the highest proportion of patients with controlled DM was seen in patients with combined DM and HTN [26].

In contrast, Type-2 DM patients almost achieved the HbA1C target at 6 months of care. This raises the question of whether a lower HbA1C target could have been reached had we considered a longer study follow-up period. Yet, a study published in Kibera, Kenya, one of the largest informal settlements in Africa, showed that there was no improvement in DM outcome beyond 6 months of care [33]. However, fasting blood glucose was used in that study as a measure for the outcome and not HbA1C. Also, the context of the Syrian crisis might have prevented patients from following lifestyle changes that are necessary to control their disease, despite education efforts by the PSEC and other health promotion activities.

The protracted Syrian crisis has increased poverty levels and probably contributed to patients’ inability to purchase appropriate food for their conditions [34]. As well, restricted movements due to lack of legal documentation [34] and the living in urban settings may have prevented Syrians from exercising properly and accessing care appropriately contributing to harder control. Increasing psychological distress [35, 36] might also have impacted their motivation to adhere to treatment plans and to follow healthy lifestyle habits.

Although LTFU in patients with DM and/or HTN is a challenge in Shatila, it was only 16%, which is better than expected considering that most of the patients came from outside the catchment area, spent time on the road before getting to the clinic, and are believed to be constantly moving and changing addresses. Retention in care in Shatila seemed less than what was observed previously in the specific context of Palestinian refugees characterized by higher stability [23, 25] but much better than what was reported in other refugee contexts where LTFU in the DM and HTN patients was observed to be as high as 40% [33, 36, 37].

While this study brings new evidence, it also has limitations. It relied on the data collected into a newly implemented, customized DHIS2 software that was somewhat difficult to implement for monitoring and data analysis. Despite the fact that the data management team was thoroughly trained on the software’s use, there was considerable missing data for some variables which prevented their use in the study, body mass index for instance. In addition, although the sample size of the outcome cohort was relatively large, it presented only 9% of the initial outcome sample for DM and 41% for HTN. Reasons were not systematically documented and hence cannot be reported. However, it could partly be due to unrecorded measurements for HbA1C and BP values in the electronic files. It could also be due to missed appointments (anecdotally around 10 NCD patients per day). Although it is possible that this sample was prone to selection bias, we have no reason to believe that it would have led to better outcomes had a different sample been selected. In fact, the characteristics of the patients who were not included in the outcome analysis were similar to the ones who did not end up being part of it on the majority of their characteristics (data not shown). Therefore, we do not believe that selection bias, if present, would have led to an important regard on our results. The use of electronic health records for an efficient disease and programmatic performance follow-up are needed and were previously recommended for such contexts [23]; strengthening routine data systems used for monitoring in the field should be a high priority. Another limitation was the 6 months follow-up for outcome assessment; it might have not been enough time to allow for stabilization of the outcome measures. Our outcome results were compared to targets that had been adapted to the humanitarian context and may not represent ideal targets for best control. In addition, our study lacks specific reporting on the frequency and severity of hypoglycemic episodes by the patients. Although information on hypoglycemia is not systematically documented in our program, patients for whom insulin will be introduced in their treatment plan, those who are on sulfonylureas or those who are in need of medication adjustments, are well educated by their doctors on the management of hypoglycemia and its risks and life threatening consequences. We do recommend for this to be followed-up in future studies specifically for patients with diabetes who are on insulin or on sulfonylureas. In addition to the above mentioned limitations, there is a possibility that our results were affected by regression to the mean. However, simulations applied on patients with more than two repeated measurements, whereby the mean of two baseline measurements was used to compare with a last measurement, did not lead to a significant change in the final conclusion reached. This study, being descriptive in nature, lacks a control group to mitigate possible regression to the mean effect and confirm the true effectiveness of the program. It does however meet the STROBE criteria for cohort studies.

## Conclusions

In conclusion, this study showed that a multidisciplinary approach to DM and HTN at the primary care level using contextualized and adapted treatment protocols was feasible in the context of a refugee camp like Shatila and achieved improved quality of care. It also showed that there might be possible to lower the treatment targets in this population to get it closer to the international guidelines. With the increasing burden of NCDs, this study suggests a comprehensive model of care for DM and HTN suitable and possibly replicable in similar contexts throughout the protracted crisis in the Middle-East and elsewhere.

## Abbreviations

ACE: angiotensin converting enzyme
BP: blood pressure
CVD: cardiovascular disease
DBP: diastolic blood pressure
DHIS2: district health information system version 2
DM: diabetes mellitus
HTN: hypertension
IQR: interquartile range
LTFU: lost to follow-up
MSF: medecins sans frontieres
NCD: non-communicable disease
NICE: national institute for health and care excellence
PSEC: patient support and education counselling
SBP: systolic blood pressure
SD: standard deviation
WHO: world health organization
